# Social Media Perspectives on a Future HIV Vaccine: Mixed Methods Analysis

**DOI:** 10.2196/82917

**Published:** 2026-03-19

**Authors:** Megan A Rabin, Sarah Penuela-Wermers, Neil K R Sehgal, Teniola I Egbe, Criswell L M Lavery, Sharath Chandra Guntuku, Alison M Buttenheim

**Affiliations:** 1 Department of Medical Ethics and Health Policy, Perelman School of Medicine, University of Pennsylvania, Blockley Hall, 423 Guardian Drive, Philadelphia, PA, 19104, United States, 1 2158987136; 2 Department of Family and Community Health, School of Nursing, University of Pennsylvania, Philadelphia, PA, United States; 3 Computer and Information Science Department, University of Pennsylvania, Philadelphia, PA, United States; 4 Leonard Davis Institute of Health Economics, University of Pennsylvania, Philadelphia, PA, United States; 5 Philadelphia VA Medical Center, Philadelphia, PA, United States

**Keywords:** HIV vaccines, vaccine hesitancy, social media, misinformation, health communication, public opinion, conspiracy theories, attitude to health, qualitative research, natural language processing

## Abstract

**Background:**

As the prospect of an HIV vaccine nears reality, understanding public discourse around the vaccine is essential for informing communication strategies and addressing misinformation. Social media platforms are influential spaces where public narratives form, yet little research has examined discourse around an HIV vaccine, especially on TikTok.

**Objective:**

This study aims to compare and characterize public discourse about a future HIV vaccine across Twitter (subsequently rebranded X) and TikTok, identifying prevailing themes, sentiments, and rhetorical strategies to inform public health communication.

**Methods:**

From over 400,000 tweets and 65,000 TikTok comments, we analyzed the 1000 most-liked posts on each platform using natural language processing and coded the top 500 most-liked posts for rhetorical strategies, sentiment, and themes.

**Results:**

Our findings reveal expressions of hope and trust in science on both platforms, as well as concerns about institutional corruption and conspiracy theories, such as the belief that the HIV vaccine responds to harm caused by the COVID-19 vaccine. Tweets tended to be more linguistically complex and yielded richer insights, while TikTok comments were shorter and more difficult to interpret without video context. Key rhetorical strategies included conspiracy theories, post hoc reasoning, and emotional appeals.

**Conclusions:**

This study underscores the need for platform-specific communication strategies to address misinformation and build public trust. The findings offer timely insights into emerging HIV vaccine discourse and highlight actionable opportunities for public health stakeholders to build trust and combat misinformation in advance of the vaccine rollout.

## Introduction

Worldwide, “infodemics,” or an oversaturation of information regardless of accuracy, have been well-documented as a threat to public health [1]. In particular, the spread of vaccine-related misinformation has emerged as a barrier to vaccine demand, acceptance, and uptake [2-4]. For example, study participants in the United States and the United Kingdom who were exposed to online COVID-19 misinformation reported decreased intention to receive a COVID-19 vaccination, including a 6 percentage point decrease among those who had previously reported they would “definitely” get the vaccine relative to those in the control group [2].

Given the volume of influential narratives and misinformation circulating on social media, it is valuable for scientists, government bodies, and public health professionals to understand public attitudes, beliefs, and concerns regarding existing and potential vaccines [1,2,4-7]. Prior studies have sought to characterize prevailing vaccine sentiment in online discourse about COVID-19, human papillomavirus, influenza, HIV, and measles vaccines, across multiple social media platforms [5-8].

Notably sparse in the online vaccine discourse literature is the HIV vaccine. This emerging vaccine, which has been the subject of recent online discussion, has over 20 clinical trials currently underway [9]. A future HIV vaccine has the potential to help end the epidemic in highly impacted regions such as Sub-Saharan Africa, home to two-thirds of the world’s population living with HIV [10]. The vaccine could also help prevent HIV in groups at the highest risk of infection, including injection drug users, sex workers, men who have sex with men, and vulnerable groups like young people and women [11].

Various studies have explored social media discourse around other aspects of HIV, namely, pre-exposure prophylaxis (PrEP) across platforms including Instagram, TikTok, Twitter (subsequently rebranded X), and Reddit [12-15]. Social media has been a place where individuals seek and share information regarding PrEP, including costs, availability, and resources [12,13,15]. Post-COVID-19, social media revealed how the pandemic disrupted various HIV prevention services, especially for racial and sexual minority groups [15]. However, social media has also served to perpetuate misinformation related to HIV [14,15]. For example, a study examining TikTok found themes about PrEP encouraging risky sex, reiterating outdated ideas about gay men as “unsafe,” and discussing HIV as a disease with poor prospects for individuals once they contract it [14].

On social media, vaccine misinformation can be especially pervasive, as messaging and communities devoted to antivaccine beliefs can rapidly grow and spread across geographic and cultural boundaries [16]. One study tracking misinformation during the COVID-19 infodemic categorized upwards of 2000 reports of “rumors” across media and social media sites, as well as another 200 reports of conspiracy theories or stigma [1]. Another study identified the increased polarization of vaccination content on Facebook, resulting in “echo chambers” in which a user might be surrounded by exclusively positive or negative vaccine attitudes depending on their interactions [17].

Despite the growing body of research studying social media attitudes regarding vaccines, only 1 study of which we are aware has specifically examined attitudes on social media regarding a future HIV vaccine, and this study excluded the social media platform TikTok [7]. Given TikTok’s rapid rise as the fastest-growing social media app in the world, with 1.5 billion active monthly users who spend an average of 52 minutes per day on the app, this is an important gap [18].

Nested within a larger trial investigating the use of messaging to “inoculate” adolescent girls and young women from HIV vaccine misinformation, this study seeks to illuminate the information ecology and characterize the discourse surrounding a future HIV vaccine across the platforms Twitter and TikTok. Both platforms offer fast-moving, public-facing communication among users, with algorithms predicting what users will want to see in an endless stream of content [19,20]. To compare the nature of the discourse across these platforms, we selected comparable, short-text posts available on the platforms: tweets on Twitter, which have a limit of 280 characters, and comments on TikTok videos, which online reports suggest had a limit of 150 characters during the time of the scrape [21-23]. We aim to highlight key insights and opportunities for action *before* the HIV vaccine is approved and available, in the hope of supporting future public health and demand creation communications.

## Methods

### Data

We scraped tweets and TikTok video comments using platform-specific protocols, using slightly different search terms due to differences in audience, purpose, and tone of content across the 2 platforms. In March 2023, we used the Twitter application programming interface and conducted a keyword-based search of relevant terms (“hiv vaccine,” “hiv vax,” “hivvaccine,” and “aids vaccine”) to collect tweets posted between January 2022 and March 2023 (a window during which 3 National Institutes of Health–funded HIV vaccine trials were in progress) [24].

Comments on TikTok videos were collected via a 2-step process. We first searched TikTok videos on October 27, 2023, using the terms “HIV Vaccine,” “AIDS Vaccine,” and “HIV Jab,” yielding a total of 777 videos. The TikTok-provided research application programming interface was used to retrieve all comments posted to those videos on or before October 31, 2023.

### Quantitative Analysis

We used the Python-based (Python Software Foundation) natural language processing software “TextAnalyzer” for lexical analysis of a subset of the high-engagement tweets and comments comprising the top-liked 1000 TikToks and 1000 tweets [25]. Posts in this dataset had a minimum of 75 likes for a given TikTok comment, or 77 likes for a tweet. TextAnalyzer provides scores on the following metrics for each unit of text input: *Flesch-Kincaid Grade Level* for sentence complexity, *Emotionality*, *Emotion* for a variety of different emotions, *Positive* and *Negative Sentiment,* for the degree of positive versus negative emotions separately, and *Emotional Valence* for a composite score indicating a positive, negative, or neutral attitude [26-30]. To understand differences in the discourse about the HIV vaccine on TikTok versus Twitter, we compared these metrics across the 2 platforms using the Welch 2-sample 2-tailed *t* tests for mean scores (conducted using R version 4.5.1; R Foundation for Statistical Computing).

### Qualitative Analysis

We coded a further subset of the top 500 most highly-engaged posts by like count, in which TikTok comments had 206 or more likes and tweets had 173 or more likes. The coders first familiarized themselves with the data and then developed a codebook including both *a priori* and emergent codes related to comment or tweet content. Based on the goals of the parent study, *a priori* codes included rhetorical and persuasion strategies from the social media and communications literatures; the veracity or validity (ie, “truth”) of any claims or information presented about the HIV vaccine, and the sentiment of the comment or tweet, that is, whether it was generally provaccine, antivaccine, or neutral. *A priori* rhetorical strategies consisted of the invocation of conspiracy theories, humor, fake experts, or highly emotional language (pathos), as well as using only selective data (cherry picking), attacking the person making an argument (ad hominem), and misattributing events happening in sequence to being cause-and-effect (post hoc propter hoc) [31,32]. We also included the strategy of “clickbait” after seeing how many posts used inflammatory and exaggerated language to fuel their argument, and “red herring” for posters who made irrelevant comments to distract the audience from the issue at hand [33]. Other sections of the codebook included “vaccine sentiment” to capture attitudes toward vaccines, “topic” for broader categories such as HIV vaccine- or COVID-19–related content, and “content themes” for more specific ideas expressed about the HIV vaccine and other emerging ideas (Multimedia Appendix 1).

This first codebook was applied to a 20% sample (100 TikTok comments and 100 tweets) of the dataset by 2 coders (MAR and SPW) to assess codebook appropriateness and establish intercoder reliability. After the initial sample coding, the coders met to iteratively revise the draft codebook, coding an additional 10% sample of the final dataset (50 TikTok comments and 50 tweets) before finalizing the codebook. Once this final codebook was agreed upon by both coders, it was applied to the entire dataset of the most-liked 500 comments and 500 tweets. The coders checked in multiple times throughout this process to resolve uncertainties in coding and ensure that they were in agreement about code applications.

After coding was complete, we analyzed a subset of 232 posts (215 tweets and 17 TikTok comments) deemed the “high-relevance” dataset because we could confidently code them to the topic area of “HIV Vaccine” across the thematic areas and rhetorical strategies outlined in the codebook. These posts were those that referenced an HIV vaccine either directly or indirectly and left minimal ambiguity or question of whether they referenced another topic of vaccine. For example, a tweet like “This has been in the works for ages but the covid stuff allowed them to accelerate mRNA research” was determined to be referencing an mRNA HIV vaccine. However, a comment reading “They’ve reached a new level of crazy…it’s actually dangerous that they’re spreading stuff like this” may have been in response to HIV vaccine discourse, but it also could have been referencing any number of other related topics such as HIV, COVID-19, or other vaccines—themes that came up in other excluded posts. Within this dataset, we identified the most prevalent topics referenced and rhetorical strategies used, and used inductive analysis to describe the themes that emerged in these posts.

### Ethical Considerations

The study was approved by the ethics committees at the University of the Witwatersrand (reference: 230904), the University of Pennsylvania (854420), the University of Cape Town (666/2023), and Boston University (H-44422). The study used only secondary data and removed usernames and identifying details from posts to preserve individual privacy.

## Results

### Dataset Description

**Figure 1.**
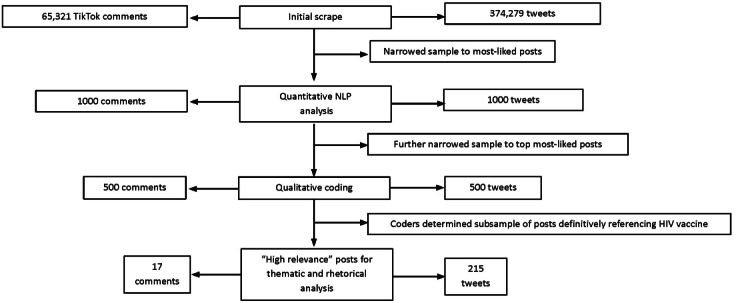
Outputs at different stages of analysis. NLP: natural language processing.

The data scrape yielded a dataset of 374,279 tweets and 65,321 TikTok comments, along with accompanying metadata. Notably, in the case of the TikTok videos, 150 out of 459 videos were posted from a single account, although we were not able to trace comments back to their original video. From these data, we conducted a quantitative analysis on the 1000 highest-liked posts from each dataset and a qualitative analysis of the top 500 posts (see Figure 1 for a diagram of the study outputs). The sections below outline the results of the lexical analysis, as well as the rhetorical and thematic analysis of the high-relevance subset of 232 posts referencing an HIV vaccine.

### Quantitative Analysis

Lexical analysis offered insight into areas in which the datasets converged and diverged (Table 1). Tweets tended to be longer than TikTok comments (41.2, SD 16.2 vs 15.7, SD 9.6 words on average; *P*<.001), with more complex sentence structure and language (16.4 vs 5.3 Flesch-Kincaid grade level score; *P*<.001).

**Table 1.** Comparative lexical analysis of tweets and TikTok comments.

Category	Range	TikTok comments (n=1000), mean (SD)	Tweets (n=1000), mean (SD)	*t* test (*P* value)
Word count	—^a^	15.8 (9.6)	41.2 (16.2)	42.5^b^ (<.001)
Flesch-Kincaid Grade level	0+	5.3 (4.7)	16.4 (5.2)	50.2^b^ (<.001)
Emotionality	0 to 9	1.8 (2.7)	1.5 (2.3)	–2.3^b^ (.02)
Emotional valence	0 (highly negative attitude) to 9 (highly positive attitude)	1.8 (3)	1.6 (2.7)	–2.0 (.05)
Anger	0 to 1	0.14 (0.12)	0.19 (0.1)	11.4^b^ (<.001)
Disgust	0 to 1	0.25 (0.23)	0.44 (0.22)	18.9^b^ (<.001)
Fear	0 to 1	0.17 (0.2)	0.29 (0.21)	13.1^b^ (<.001)
Joy	0 to 1	0.25 (0.29)	0.30 (0.23)	4.4^b^ (<.001)
Anticipation	0 to 1	0.19 (0.22)	0.32 (0.21)	13.2^b^ (<.001)
Trust	0 to 1	0.26 (0.35)	0.37 (0.26)	7.9^b^ (<.001)
Negative sentiment	−1 to 0	−0.37 (0.22)	−0.42 (0.17)	−5.0^b^ (<.001)
Positive sentiment	0 to 1	0.29 (0.26)	0.3 (0.12)	−0.4 (.72)

a Not applicable.

b significant at *P*<.05.

Tweets showed higher mean scores than comments for all 6 measured emotions, with markedly greater differences for disgust (0.44, SD 0.22 in tweets; 0.25, SD 0.23 in comments; *P*<.001), anticipation (0.32, SD 0.21 in tweets; 0.19, SD 0.22 in comments; *P*=<.001) and fear (0.29, SD 0.21 in tweets; 0.17, SD 0.2 in comments; *P*<.001). In terms of emotionality, TikTok comment scores were only slightly higher (0.25 difference on the 0‐9 score; *P*=.02). This indicates that the sentiments expressed in TikTok comments were more heavily based in emotion than tweets, though this did not necessarily map onto specific emotions generated by TextAnalyzer. Additionally, the two were similar in terms of mean emotional valence (1.8, SD 3 for comments and 1.6, SD 2.7 for tweets; *P*=.05), tending toward negative attitudes, though comments were slightly more positive. This was also evident in sentiment; the platforms had somewhat similar mean negative sentiment scores (−0.42, SD 0.17 for tweets; −0.37, SD 0.22 for comments; *P*<.001) slightly greater than their mean positive sentiment score (0.3, SD 0.12 for tweets; 0.29, SD 0.26 for comments; *P*=.72). This showed that posts exhibited moderate average positive and negative associations, skewing slightly higher toward negative sentiment, and with incrementally greater averages for tweets versus comments.

### Qualitative Analysis

Coders examined the discourse surrounding HIV vaccine development on TikTok and Twitter. The codes fell into 4 categories: topic, vaccine sentiment, content themes, and rhetorical strategies. Out of 500 tweets and 500 TikTok comments, 215 tweets and 17 TikTok comments were coded under the topic code “HIV vaccines.” The themes and rhetorical strategies most prevalent in this highly relevant dataset of 232 total posts are outlined below. See Table 2 for a full list of key themes and rhetorical strategies.

**Table 2.** Key themes and rhetorical strategies.

	Definition	Example
Key theme		
Scientific and technological progress	Displays of trust and celebration of scientific research and technological progress, as evidenced by HIV vaccine development.	“...This is really exciting news I wanted to share. The first participant has been dosed in the phase I study of Moderna’s HIV vaccine candidate, mRNA-1644, which uses the same mRNA technology as our COVID-19 vaccines!” [Tweet, record #7]
Hope and excitement for HIV vaccine	Enthusiasm voiced for an HIV vaccine and the impact it may hold.	“Wow—as a queer elder, this news made me tear up: An HIV vaccine is in phase 1 trials!!!” [Tweet, record #184]
Distrust in pharmaceutical companies and government	Suspicion of the government’s and pharmaceutical companies’ motives as fueled by news of HIV vaccine development	“Any guess on how long it will be before your rights of free movement are restricted until you have taken the new HIV vaccine? I mean, we can’t be too careful ‘for the common good…’*”* [Tweet, record #272]
Belief that COVID-19 vaccine causes HIV	Ideas that recent progress in HIV vaccine development confirms the conspiracy theory that the COVID-19 vaccine causes HIV	“So when the shot causes VAIDS and you test positive for HIV don’t panic!! They’ve got you covered with an experimental mRNA vax to make you all better 🤣” [Tweet, record #462]
Key rhetorical strategy		
Pathos	Use of vivid, emotional language in describing excitement and hope regarding an HIV vaccine	“If this came 6 yrs ago I might still have [name]. He is loved and missed. There is so much hope, I’m crying happy tears” [TikTok comment, record #380]
Conspiracy	Depicting HIV vaccine development as an indicator of a secret, often malevolent plot in order to evoke mistrust in an entity (ie, the government, pharmaceutical industry)	“There is no vaccine for HIV after 40 years of research. No cure for cancer after 100 years of research. The flu spreads like wildfire each year without any concern. Yet a virus appears out of nowhere, a vaccine magically appears from 4 different pharma and forced on us?.” [Tweet, record #10]
Key figures	Invoking prominent figures to garner support for or against HIV vaccines	“Groundhog Day: Nearly every year since 1983, Fauci extracted another billion+from Congress promising AIDS Vax just as the press dolefully announces failure of his most recent candidate. His Rasputin-like hold on press+politicians means game never ends.” [Tweet, record #23]
Post hoc propter hoc	Fallacy that events happening in succession must have a causal relationship, namely with regard to fears that the COVID-19 vaccine causes HIV, and subsequent strides in HIV vaccine development	“Last week it was announced there was a new aggressive HIV going around in Neatherlands [sic], the next day [it] was announced there is a new MRNA HIV shot. Create a problem (HIV in vax) then create the solution (new HIV vax). Just our world leaders doing what they do” [Tweet, record #137]

### Content Themes

#### Scientific and Technological Progress (57 Posts)

Content demonstrated trust in scientific research and celebration of technological advancements, particularly in the realm of vaccine development, across a total of 57 posts. Fifteen posters specifically highlighted advancements in mRNA technology during COVID-19 vaccine development, and how these advancements have helped bring the HIV vaccine closer to reality:

*...This is really exciting news I wanted to share. The first participant has been dosed in the phase I study of Moderna’s HIV vaccine candidate, mRNA-1644, which uses the same mRNA technology as our COVID-19 vaccines!*

[Tweet, record #7]

#### Hope and Excitement for the HIV Vaccine (40 Posts)

Posters expressed optimism regarding the development of an HIV vaccine in 40 posts, and even described it as a monumental breakthrough. The vaccine was seen as offering hope, especially to those who have been personally impacted by HIV, and as a critical step forward in the HIV response:

*Wow—as a queer elder, this news made me tear up: An HIV vaccine is in phase 1 trials!!!*

[Tweet, record #184]

#### Distrust in Pharmaceutical Companies and Government (34 Posts)

Many posters expressed a lack of trust in pharmaceutical companies and the government. There was speculation that the “sudden” announcement of HIV vaccine trials after mass COVID-19 vaccinations was part of a larger conspiracy—whether to cover up COVID-19 vaccine–induced HIV or to exploit and control the public. Posters also voiced concerns about government support for an HIV vaccine. They feared that they would be forced to take the vaccine against their will, as they felt had happened with COVID-19 vaccine mandates:

*Any guess on how long it will be before your rights of free movement are restricted until you have taken the new HIV vaccine? I mean, we can’t be too careful ‘for the common good…’*

[Tweet, record #272]

#### Belief That COVID-19 Vaccine Causes HIV (20 Posts)

Various tweets and comments used HIV vaccine development announcements to amplify posters’ theories that the COVID-19 vaccine causes HIV. These posters viewed the HIV vaccine development as proof of a conspiracy, claiming that pharmaceutical companies developed COVID-19 vaccines to infect recipients with HIV and thereby increase demand for their HIV vaccines.

*So when the shot causes VAIDS and you test positive for HIV don’t panic!! They’ve got you covered with an experimental mRNA vax to make you all better* 🤣

[Tweet, record #462]

### Common Rhetorical Strategies

#### Overview

Below are the most common rhetorical strategies, listed in order of frequency. We included the 4 most frequent rhetorical strategies because the second to fourth most-coded strategies appeared nearly the same number of times in the sample, with 20 to 22 references, after which point rhetorical strategies dropped to 15 or fewer codes in the high-relevance sample.

#### Pathos (27 Posts)

Some posters expressed heightened emotion, or pathos, in their posts. These 27 posts were overwhelmingly pro–HIV vaccine; for example, posters shared their joy for a vaccine and the sadness about the toll of HIV so far. In describing their personal hopes, struggles, and celebrations, these posts draw posters into more personal narratives supporting HIV vaccines and invite them to feel delight in the progress or grief for lives lost alongside the original poster.

*If this came 6 yrs ago I might still have [name]. He is loved and missed. There is so much hope, I’m crying happy tears*

[TikTok comment, record #380]

#### Conspiracy (22 Posts)

Conspiracy theories emerged as a predominant rhetorical strategy used by anti–HIV vaccine posters. In these comments and tweets, posters conjured up and repeated extreme and dire circumstances as proof that the HIV vaccine cannot be trusted. By making users fear the most horrific of circumstances, posters generated doubt in the safety of the HIV vaccine and those producing it. As described in the “Pathos” section, various posters parroted the conspiracy theory that the COVID-19 vaccine causes HIV. They were convinced that the government and pharmaceutical companies were being duplicitous in hiding this and trying to promote an HIV vaccine in response to “vaccine-induced AIDS.” Some were also convinced that the speed with which the COVID-19 vaccine was developed indicated a kind of government and pharmaceutical conspiracy.

*There is no vaccine for HIV after 40yrs of research. No cure for cancer after 100yrs of research. The flu spreads like wildfire each year without any concern. Yet a virus appears out of nowhere, a vaccine magically appears from 4 different pharma and forced on us?....*

[Tweet, record #10]

#### Key Figures (21 Posts)

Various posters mentioned key figures in their posts regarding an HIV vaccine. In this strategy, posters referenced well-known individuals to support their (largely anti-HIV vaccine) arguments—figures whom users would easily recognize. By naming these individuals, most of whom are powerful and evoke strong opinions, posters attach a face to the corruption or mistrust they are trying to incite.

One figure who was often mentioned to support users’ distrust of the government and vaccines was Anthony Fauci, former director of the National Institute for Allergy and Infectious Diseases, who has been a central figure in the HIV and COVID-19 responses [34]. Twelve posters pointed to past statements by Fauci about HIV vaccine development, using them to foster distrust in current vaccine development practices. Other key figures mentioned include Prince Harry, Elon Musk, and Luc Montagnier, who was awarded the Nobel Prize for his discovery of HIV and later perpetuated antivaccine conspiracy theories in his later years [35].

*Groundhog Day: Nearly every year since 1983, Fauci extracted another billion+from Congress promising AIDS Vax just as the press dolefully announces failure of his most recent candidate. His Rasputin-like hold on press+politicians means game never ends.*

[Tweets, record #23]

#### Post Hoc Propter Hoc (20 Posts)

Twitter and TikTok posters often use post hoc propter hoc reasoning (assuming that because one event happened after another, they must have a causal relationship) to convince readers that the HIV vaccine cannot be trusted. By pairing the 2 events together (eg, mass COVID vaccination and announcements of new HIV vaccine clinical trials), the posters suggest or explicitly tell readers that the two must be related, and that there is a sort of causal relationship between them.

*Last week it was announced there was a new aggressive HIV going around in Neatherlands [sic], the next day [it] was announced there is a new MRNA HIV shot. Create a problem (HIV in vax) then create the solution (new HIV vax). Just our world leaders doing what they do.*

[Tweet, record #137]

## Discussion

### Principal Findings

In this study, we conducted a mixed-methods comparative, thematic, and rhetorical analysis of public discourse surrounding the HIV vaccine on Twitter and TikTok. We focused on the most highly engaged posts, first selecting the 1000 most-liked for quantitative analysis, then the 500 most-liked for qualitative analysis. We found that the majority of content in direct reference to the HIV vaccine came from Twitter, and that tweets tended to be longer and more linguistically complex than TikTok comments. This may in part be due to the different character limits between the apps—280 characters for tweets versus 150 characters for TikTok comments [21-23]. Major thematic areas included support for scientific progress and excitement around vaccine development, as well as distrust in government and scientific entities, and beliefs that the HIV vaccine is being introduced to combat COVID-19 vaccine–induced HIV. Across both platforms, there was a mix of positive and negative sentiment regarding HIV vaccines, and a pattern of rhetorical strategies used to convey these ideas. The dichotomy of emotions toward the HIV vaccine was evident; emotions most seen in tweets were disgust and trust, and in TikTok comments were trust, joy, and disgust.

We aimed to analyze not only what people were saying about an HIV vaccine online, but also how they were saying it. Using *a priori coding* informed by misinformation research, we identified and refined four common rhetorical strategies: conspiracy theories, post hoc propter hoc, references to key figures, and pathos. The first three strategies (conspiracy theories, post hoc reasoning, and references to key figures) were primarily associated with anti-HIV-vaccine sentiment. For example, some posters claimed that the HIV vaccine was being pushed as a response to the COVID-19 vaccine supposedly infecting people with HIV, suggesting government or pharmaceutical conspiracies and citing figures like Anthony Fauci as evidence of corruption. In contrast, pathos was more common in provaccine messaging. Supporters shared personal stories of loss during the HIV pandemic and expressed hope and excitement at the prospect of a vaccine. These emotionally resonant narratives appeared to foster more positive perceptions of the HIV vaccine. Understanding both negative and positive rhetorical strategies provides insight into how vaccine discourse unfolds online and highlights potential pathways for encouraging public support.

In our literature search, we found only 1 study pertaining to social media discourse on HIV vaccines specifically. In this 2024 study, Zhang et al [7] used machine learning algorithms to analyze tweets posted in 2022 about HIV vaccines, then manually coded the most highly engaged tweets about HIV and COVID-19. This study situated the discourse surrounding the HIV vaccine largely within the context of the COVID-19 vaccine, similarly revealing both positive sentiments as posters compared the progress of one vaccine to another, and negative sentiments as they parroted the conspiracy theory that the COVID-19 vaccine causes HIV, and the introduction of an HIV vaccine as part of a larger government conspiracy.

Other studies in related topic areas indicate similar thematic trends. One study of antivaccine attitudes on social media found an emphasis on conspiracy theories in messaging, complete with “secret, sinister organizations and manipulative government bodies causing harm” [36]. In this sense, the perpetuation of conspiracy theories is not unique to the topic of HIV vaccines, although the topic of those theories may vary from vaccine to vaccine. A study of COVID-19 vaccine social media discourse also showed a mixture of positive sentiment about vaccine development and availability, combined with negative sentiments about vaccine safety, conspiracies, and the impact of the government [5].

The themes uncovered in our HIV vaccine discourse analysis are distinct from those in past studies of PrEP communication on social media. Broadly, users sought to share information about both topics, and topics in both areas included misinformation about HIV and its prevention [12,13,15]. However, there were different conversations about who is most impacted by HIV. A TikTok study of PrEP communication found that homophobic and heteronormative misconceptions influenced much of the dialogue [14]. This theme was not present in our data, where conversations focused more on the hope of an HIV vaccine after losing so many people in the queer community to HIV. This is interesting, considering that both the HIV vaccine and PrEP are designed to prevent HIV. However, the differences in discussion may be due to the fact that there is far less concrete information about the HIV vaccine at this stage than there is about PrEP.

During the qualitative analysis, it became clear that most data confidently categorized as related to an HIV vaccine came from Twitter. A total of 248 TikTok comments, nearly half the sample, were coded as “unable to ascertain,” compared to only 10 tweets, indicating difficulty in determining the subject matter of many TikTok comments. Vague remarks like “This is the kind of history I want to live through” or “my jaw dropped” lacked clear topic indicators and offered limited qualitative insight. One reason for this may be that tweets often stand alone and convey meaning independently, whereas TikTok comments rely heavily on the accompanying video, making interpretation difficult without that context. This issue was compounded by different scraping methods: tweets entered the dataset only if they included specific search terms, while TikTok comments were collected if they responded to videos that matched search criteria, regardless of their own content.

Furthermore, the average TikTok comment contained 15.8 (SD 9.6) words, nearly a third of the tweets’ average of 41.2 (SD 16.2) words. In addition, the comments’ Flesch-Kincaid grade level was an average of 5.3 (SD 4.7), much lower than the tweets’ estimated average of 16.4 (SD 5.2). In other words, one dataset is composed of fairly short, simplistic sentiments, while the other contains much longer ideas written at a graduate level. As such, it is perhaps unsurprising that the tweets yielded richer data regarding an HIV vaccine.

This study’s findings can inform public health messaging by highlighting the distinct communication needs of social media audiences across platforms. Murthy et al’s [37] “3 Rs” framework—reviewing the audience, recognizing communication needs, and responding appropriately—offers a useful model for time-sensitive health topics like the HIV vaccine. Our analysis supports the first 2 “Rs” by identifying audience communication patterns, prevalent themes, and rhetorical strategies. We found that TikTok and Twitter posters’ most prevalent barriers to HIV vaccine acceptance are a lack of trust in the government and scientific institutions, as well as the belief in the conspiracy theory that the HIV vaccine was introduced as a response to the COVID-19 vaccine–induced HIV. Additionally, many people *do* feel excited about the idea of future HIV vaccines, and many cited personal stories and emotions about the lifesaving potential of an HIV vaccine. Should future public health campaigns arise to combat HIV vaccine misinformation, they might focus on rebuilding trust in the scientific and governmental systems, dispelling misinformation about COVID-19 vaccine–induced HIV, and using personal stories from real people to show the benefits an HIV vaccine offers. Effective messaging should also consider platform differences, using more scientific, complete sentences on Twitter and shorter, simpler language on TikTok to better engage each audience.

### Limitations

This study offers a snapshot of evolving social media discourse on the HIV vaccine and focuses only on English-language posts. Since dialogue on social media moves much faster than the research following it, these data do not capture trends or events occurring from 2024 to the present. However, we feel confident that the core themes extracted from TikTok and Twitter remain relevant and beneficial to researchers and policymakers in the HIV vaccine space. In addition, TikTok and Twitter are 2 distinct platforms with different post offerings, which pose a challenge in comparing their content. Although the study considers short text posts on both platforms, the tweets analyzed were a product of direct keyword filtering, whereas TikTok comments came from videos matching the search terms. As such, stand-alone TikTok comments largely offered less insight than tweets and made up a much smaller proportion of the high-relevance dataset. For this reason, we also extended the window of time over which TikTok comments could be posted to include in the study, ranging until October 2023, about 7 months later than the tweets we included. While the results give new insights into an uncharted area of HIV vaccine discourse on social media, the limitations in the comparative aspect of the qualitative analysis are important to consider. It should also be noted that nearly one-third of TikTok videos from which comments were scraped came from a single account that reposted content from others. Since there was no significant alteration in these reposts, we find that the comments scraped still reflect prevailing TikTok narratives about the HIV vaccine. However, we acknowledge that the followers of this account may be more heavily represented in our findings.

### Directions for Further Research

At the time of publication, there is little research analyzing TikTok comments or comparing TikTok comments to other social media posts. Further research should aim to illuminate best practices in analyzing this type of information and extracting themes from TikTok comments. Future research should also continue to examine the discourse surrounding the HIV vaccine, especially as trials progress, on different social media platforms. Other platforms such as Instagram, or the newly popular platform BlueSky—a more liberal substitute for Twitter for many—may offer a new lens on HIV vaccine discourse to compare to other platforms [38]. Researchers should also consider piloting messaging or interventions to curb misinformation surrounding the vaccine, especially if it involves penetrating online groups that are echo chambers for vaccine distrust and misinformation.

### Conclusions

As medical technology advances, the prospect of a successful HIV vaccine feels closer than ever. Thus, it is essential to understand the nature of public discourse regarding the vaccine. By conducting a mixed methods comparative analysis of Twitter and TikTok comments, we offer further context and additional themes in this exceedingly small body of literature. These insights are valuable to public health, medical, and governmental actors seeking to promote accurate information and foster trust in the HIV vaccine. Our findings show that while both platforms feature expressions of hope and trust in science, they also contain concerns about institutional corruption and conspiracy theories, such as the belief that the HIV vaccine responds to harm caused by the COVID-19 vaccine. Tweets tended to be more linguistically complex and yielded richer insights, while TikTok comments were shorter and more difficult to interpret without video context. Key rhetorical strategies included conspiracy theories, post hoc reasoning, and emotional appeals. These findings underscore the need for platform-specific communication strategies to address misinformation and build public trust. As conversations on social media continue to rapidly evolve, future research should continue to monitor and analyze new trends in sentiment toward HIV vaccines. Researchers should also broaden the scope to include additional text-based platforms such as Threads, as well as other languages, and explore tailored interventions that align with each platform’s communication style. By understanding how users express themselves in the midst of misinformation and an ever-changing social media landscape, we can craft more empathetic, accurate, and effective public health messaging to support confidence in an HIV vaccine.

## Supplementary material

10.2196/82917 Multimedia Appendix 1 Codebook developed by coders to qualitatively analyze tweets and comments.

## Abbreviations

PrEP: pre-exposure prophylaxis

## References

[1] Islam MS, Sarkar T, Khan SH (2020). COVID-19–related infodemic and its impact on public health: a global social media analysis. Am J Trop Med Hyg.

[2] Loomba S, de Figueiredo A, Piatek SJ, de Graaf K, Larson HJ (2021). Measuring the impact of COVID-19 vaccine misinformation on vaccination intent in the UK and USA. Nat Hum Behav.

[3] Burki T (2019). Vaccine misinformation and social media. Lancet Digit Health.

[4] Roozenbeek J, Schneider CR, Dryhurst S (2020). Susceptibility to misinformation about COVID-19 around the world. R Soc Open Sci.

[5] Hussain A, Tahir A, Hussain Z (2021). Artificial intelligence-enabled analysis of public attitudes on Facebook and Twitter toward COVID-19 vaccines in the United Kingdom and the United States: observational study. J Med Internet Res.

[6] Wawrzuta D, Jaworski M, Gotlib J, Panczyk M (2021). Characteristics of antivaccine messages on social media: systematic review. J Med Internet Res.

[7] Zhang JM, Wang Y, Mouton M, Zhang J, Shi M (2024). Public discourse, user reactions, and conspiracy theories on the X platform about HIV vaccines: data mining and content analysis. J Med Internet Res.

[8] Basch CH, MacLean SA (2019). A content analysis of HPV related posts on Instagram. Hum Vaccin Immunother.

[9] (2025). HIV vaccines. International AIDS Vaccine Initiative.

[10] Moyo E, Moyo P, Murewanhema G, Mhango M, Chitungo I, Dzinamarira T (2023). Key populations and Sub-Saharan Africa’s HIV response. Front Public Health.

[11] (2025). Vulnerable groups and key populations at increased risk of HIV. World Health Organization Regional Office for the Eastern Mediterranean.

[12] Edinger A, Valdez D, Walsh-Buhi E, Bollen J (2023). Deep learning for topical trend discovery in online discourse about pre-exposure prophylaxis (PrEP). AIDS Behav.

[13] Walsh-Buhi E, Houghton RF, Lange C, Hockensmith R, Ferrand J, Martinez L (2021). Pre-exposure prophylaxis (PrEP) information on Instagram: content analysis. JMIR Public Health Surveill.

[14] Lewis J, Melendez-Torres GJ (2024). Prep-Tok: a queer critical discourse analysis of TikToks regarding HIV-related pre-exposure prophylaxis. Cult Health Sex.

[15] Xu Q, McMann T, Godinez H (2023). Impact of COVID-19 on HIV prevention access: a multi-platform social media infodemiology study. AIDS Behav.

[16] Muric G, Wu Y, Ferrara E (2021). COVID-19 vaccine hesitancy on social media: building a public Twitter data set of antivaccine content, vaccine misinformation, and conspiracies. JMIR Public Health Surveill.

[17] Schmidt AL, Zollo F, Scala A, Betsch C, Quattrociocchi W (2018). Polarization of the vaccination debate on Facebook. Vaccine (Auckl).

[18] Woodward M (2021). TikTok user statistics 2025: everything you need to know. SearchLogistics.

[19] Hern A (2022). How TikTok’s algorithm made it a success: ‘it pushes the boundaries’. The Guardian.

[20] (2023). Twitter’s recommendation algorithm. X.

[21] Counting characters. X.

[22] (2025). TikTok business capabilities and limitations. Sprinklr Help Center.

[23] TikTok character counter. CharacterCounter.com.

[24] (2022). NIH launches clinical trial of three mRNA HIV vaccines. National Institutes of Health.

[25] Berger J, Sherman G, Ungar L (2020). TextAnalyzer.

[26] Kincaid JP, Fishburne RP, Rogers RL, Chissom BS (1975). Derivation of new readability formulas (automated readability index, fog count and Flesch reading ease formula) for navy enlisted personnel. https://apps.dtic.mil/sti/pdfs/ADA006655.pdf.

[27] Kiritchenko S, Zhu X, Mohammad SM (2014). Sentiment analysis of short informal texts. J Artif Intell Res.

[28] Mohammad SM #Emotional tweets. https://aclanthology.org/S12-1033/.

[29] Mohammad SM, Kiritchenko S (2015). Using hashtags to capture fine emotion categories from tweets. Comput Intell.

[30] Rocklage MD, Rucker DD, Nordgren LF (2018). The Evaluative Lexicon 2.0: the measurement of emotionality, extremity, and valence in language. Behav Res Methods.

[31] Hopkins KL, Lepage C, Cook W (2023). Co-designing a mobile-based game to improve misinformation resistance and vaccine knowledge in Uganda, Kenya, and Rwanda. J Health Commun.

[32] Roozenbeek J, van der Linden S (2019). Fake news game confers psychological resistance against online misinformation. Palgrave Commun.

[33] Da San Martino G, Barrón-Cedeño A, Wachsmuth H, Petrov R, Nakov P SemEval-2020 task 11: detection of propaganda techniques in news articles.

[34] (2025). Anthony S. Fauci, M.D. National Institute of Allergy and Infectious Diseases.

[35] Kazi F, Mushtaq A (2022). Luc Montagnier. Lancet Infect Dis.

[36] Mitra T, Counts S, Pennebaker J Understanding anti-vaccination attitudes in social media.

[37] Murthy BP, LeBlanc TT, Vagi SJ, Avchen RN (2021). Going viral: the 3 Rs of social media messaging during public health emergencies. Health Secur.

[38] Chayka K (2025). Bluesky’s quest to build nontoxic social media. New Yorker.

