# Supplementary material for: Social Media Perspectives on a Future HIV Vaccine: Mixed Methods Analysis
Source: JMIR Infodemiology. 2026 Mar 19;6:e82917. doi: 10.2196/82917 (PMC13001999; doi:10.2196/82917)
Supplement: Multimedia Appendix 1 [file infodemiology-v6-e82917-s001.docx]

Appendix:

Codebook developed by coders in order to qualitatively analyze tweets and comments:

| Name | Description |
| --- | --- |
| Containing Misinformation | Whether or not the comment/ tweet contains misinformation |
| False Information | Use when the data contains outright false information |
| Misleading | Use when data contains a statement that is not correct but is also not outright false information |
| Not Misinformation | Use when the data contains either true information or only opinions. I.e. does not contain any type of misinformation |
| Unable to ascertain | Use when the data may have true information, misinformation, or misleading information, but it is impossible to tell |
| Content Themes |  |
| Concerns about availability | Use if data expresses thoughts about the availability of an HIV Vaccine |
| Cost | Use when data mentions of financial cost of HIV to a patient including but not limited to the vaccine, treatment, or stigma |
| COVID vax causes AIDS | Use if the data expresses concern or states that the COVID vaccine can or does cause HIV/AIDS |
| Ctitical of anti-vax | Use if the data seems to critique members of or arguments used by the anti-vax community |
| Efficacy of HIV vax | Use if data expresses talks about HIV Vaccine’s efficacy, or lack thereof |
| Ethical concerns | Use if data mentions ethical concerns related to the HIV vaccine including but not limited to: the testing process and development process |
| Exposure from HIV vaccine | Use when the data expresses the concern that a HIV vaccine increasing the recipient’s exposure to HIV |
| Gov't trust | Use when data expresses trust in government or lack thereof |
| HIV in COVID vax | Use if the data mentions parts of HIV as ingredients in the COVID vaccine |
| HIV Vax trust | Use when data expresses trust in an HIV vaccine or lack thereof |
| Key figures | Use if the data references a key people or public figures in the Political scene, vaccine development, public health, or HIV movement |
| Life saving | Use if data expresses that an HIV Vaccine will save lives |
| Non-necessity | Use if data expresses the view that an HIV Vaccine is not necessary, including having other (PrEP ART or natural remedy options) |
| Pharma Trust | Use when data expresses trust in pharmaceutical companies or lack thereof |
| PLWH | People living with HIV support- use in positive references to people who are HIV+ |
| Politics | Use when the data expresses some type of political commentary or mentions a political party/ figure |
| Public Health Trust | Use when data expresses trust in public health efforts or institutions or lack thereof |
| Reflection | Use if data expresses nostalgia or references to HIV progress, or some sort of event in the past |
| Safety | Use if data expresses concerns about an HIV Vaccine’s safety |
| Science Trust | Use when data expresses trust in science or lack thereof |
| Side effects | Use if data comments on vaccine side effects |
| Stigma | Use in references to stigma regarding HIV, the HIV vaccine, or people associated with either |
| Tech advance in COVID vax | Use when the data references development of the COVID vaccine leading to advancements in vaccination in general |
| Not English | Use for codes that are not in the English language |
| Rhetorical Strategy |  |
| Ad hominem name calling | Use when data is attacking or using name-calling to attack a person or idea to discredit their argument, instead of the argument itself |
| Post hoc propter hoc | Use when data incorrectly links an exposure and an outcome that do not have a causal relationship in order to mislead others that they do. This includes causal simplification |
| Cherry picking | Use if data uses incomplete information or distorted data to paint an incorrect or misleading picture |
| Clickbait | Use when data uses extreme or flashy language to catch attention |
| Conspiracy | Use if data references or perpetuates a conspiracy theory |
| Fake experts | Use when data invokes information from or expertise of a false expert or professional |
| False dilemma | Use when data incorrectly assumes an either/or situation. Aka false dichotomy |
| Humor | Use when the data uses humor to make or emphasize their point |
| Pathos | Use if data appears to seek an emotional response through strong language, narrative, or scare tactics |
| Topic | Broader topic of the post |
| COVID | Use if data is about COVID and/or its vaccine |
| HIV | Use if data is about HIV but not an HIV Vaccine |
| HIV Vaccine | Use if data is about an HIV Vaccine |
| Linked | Comment/tweet contains a URL |
| Other | Use if data is about anything other accompanying sub-codes |
| Trial Announcement | Use if the comment/tweet is announcing something relating to an HIV Vaccine trial |
| Unable to Ascertain | Use if it is difficult or impossible to tell what the comment is referencing |
| Vaccines | Use if data is about vaccines other than HIV or COVID vaccines, or about vaccines in general |
| Vaccine Sentiment |  |
| Anti-Vaccine | Use when data takes an anti-vaccine stance |
| Anti-HIV Vaccine | Use when data expresses sentiment against an HIV Vaccine, specifically |
| Pro-Vaccine | Use when data actively supports some form of vaccination |
| Unable to ascertain | Use when data expresses a strong sentiment towards vaccines, but could be interpreted as either pro or against |
| Vaccine-Neutral | Use when data expresses neither positive nor negative views of vaccination |
